# A Comparative Study on the Mycelium and Fruiting Body of *Meripilus giganteus*: Chemical Composition and Biological Activity

**DOI:** 10.3390/cimb47050302

**Published:** 2025-04-25

**Authors:** Katarzyna Sułkowska-Ziaja, Mateusz Korczyński, Monika Trepa, Agnieszka Galanty, Jan Lazur, Paweł Kubica, Katarzyna Kała, Paweł Paśko, Bożena Muszyńska

**Affiliations:** 1 Department of Medicinal Plant and Mushroom Biotechnology, Faculty of Pharmacy, Jagiellonian University Medical College, Medyczna 9, 30-688 Kraków, Poland; monika.trepa@doctoral.uj.edu.pl (M.T.); jan.lazur@doctoral.uj.edu.pl (J.L.); p.kubica@uj.edu.pl (P.K.); k.kala@uj.edu.pl (K.K.); bozena.muszynska@uj.edu.pl (B.M.); 2 Student’s Scientific Group of Medicinal Plant and Mushroom Biotechnology, Faculty of Pharmacy, Jagiellonian University Medical College, Medyczna 9, 30-688 Kraków, Poland; mateusz.1.korczynski@student.uj.edu.pl; 3 Doctoral School of Medical and Health Sciences, Jagiellonian University Medical College, Św. Łazarza 16, 30-530 Kraków, Poland; 4 Department of Pharmacognosy, Faculty of Pharmacy, Jagiellonian University Medical College, Medyczna 9, 30-688 Kraków, Poland; agnieszka.galanty@uj.edu.pl; 5 Department of Food Chemistry and Nutrition, Faculty of Pharmacy, Jagiellonian University Medical College, Medyczna 9, 30-688 Kraków, Poland; p.pasko@uj.edu.pl

**Keywords:** antioxidant activity, biologically active compounds, cytotoxicity, gastrointestinal cancers, *Meripilus giganteus*, mycelium

## Abstract

*Meripilus giganteus* (Pers.) P. Karst. is a basidiomycete fungus known for its bioactive properties, including antioxidant, antimicrobial, and cytotoxic effects. Although research has largely focused on fruiting bodies, mycelium obtained through in vitro culture offers a sustainable and potentially scalable source of bioactive metabolites. This study aimed to compare the chemical composition and biological activity of extracts from the fruiting bodies and mycelium of *M. giganteus*. Key compound groups were analyzed using high-performance liquid chromatography (HPLC), and biological activity was assessed through DPPH and ABTS antioxidant assays and MTT-based cytotoxicity testing on human gastrointestinal cancer and normal colon epithelial cell lines. The results revealed distinct metabolite profiles between fungal forms and demonstrated that solvent type strongly influenced extraction efficiency. Cytotoxicity assays indicated moderate activity of both extract types, with some selectivity towards colorectal cancer cell lines. These findings suggest that *M. giganteus* mycelium may serve as a promising alternative to fruiting bodies for the production of antioxidant and potentially chemopreventive compounds. Further studies are recommended to optimize cultivation and extraction conditions to enhance both metabolite yield and biological activity.

## 1. Introduction

Medicinal mushrooms have attracted increasing attention due to their rich chemical composition and diverse biological activities [1,2]. *Meripilus giganteus* (Pers.) P. Karst., a basidiomycete belonging to the family Meripilaceae, is a large, wood-decaying fungus commonly found on deciduous trees in Europe with significant ecological and medicinal potential. It plays a crucial role in forest ecosystems by decomposing lignin-rich substrates and contributing to nutrient cycling [3]. Beyond its ecological function, this species has also been recognized for its bioactive properties, including antioxidant, antimicrobial, anticancer, immunosuppressive, and neuroprotective effects [4,5,6]. The presence of various secondary metabolites, including polysaccharides, sterols, cerebrosides, fatty acids, and phenolic compounds, underlies these activities, making *M. giganteus* a promising candidate for pharmaceutical and biotechnological applications [6,7,8,9]. Most studies on *M. giganteus* have focused on its fruiting bodies, which have been shown to exhibit cytotoxic effects against selected cancer cell lines, antimicrobial activity against bacterial and fungal pathogens, and the ability to inhibit acetylcholinesterase, suggesting potential applications in neurodegenerative disorders such as Alzheimer’s disease [7]. Despite these promising findings, research on its mycelium remains limited. Mycelium provides an alternative to fruiting bodies, ensuring stable and scalable growth while being independent of seasonal and environmental conditions [8]. However, due to potential differences in metabolite biosynthesis between fruiting bodies and mycelial cultures, a comparative evaluation is necessary to determine whether mycelium can serve as a viable alternative source of bioactive compounds [9].

One of the major challenges in modern medicine is the increasing prevalence of civilization-related diseases, particularly cancers of the gastrointestinal tract, which rank among the leading causes of morbidity and mortality worldwide [10]. Chronic inflammation is recognized as a critical factor in the development and progression of these malignancies, with oxidative stress playing a key role in promoting DNA damage, tumor initiation, and disease progression [11]. Antioxidants, which neutralize reactive oxygen species (ROS), have been extensively studied for their potential in cancer prevention and therapy, particularly in reducing inflammation-induced carcinogenesis. Mushrooms are a valuable source of bioactive compounds with potential therapeutic applications in civilization-related diseases, including gastrointestinal cancers [11]. Given the previously reported antioxidant activity of *M. giganteus*, further research is necessary to elucidate its potential role in counteracting oxidative stress, particularly in the context of gastrointestinal cancers.

This study aims to address this gap by analyzing and comparing the chemical composition and biological activity of extracts derived from the fruiting bodies and mycelial cultures of *M. giganteus*. The key hypothesis underlying this research is that mycelium can produce comparable or even higher levels of bioactive metabolites than fruiting bodies, making them a sustainable alternative for obtaining medicinally valuable compounds. To test this hypothesis, chromatographic techniques are applied to identify and quantify various metabolite groups, including sterols, indole derivatives, proteinogenic amino acids, phenolic acids, statins, and sulfur-containing antioxidants. Additionally, in vitro assays are conducted to assess the cytotoxic effects of different extracts on a panel of human gastrointestinal cancer cell lines (DLD-1, HCT116, HT29, HepG2), as well as a non-cancerous human colon epithelial cell line (CCD 841 CoN). The inclusion of this non-cancerous cell line enables the evaluation of the selectivity of the extracts’ cytotoxic activity, which is crucial for potential therapeutic applications. Given that oxidative stress is a major contributor to cancer progression, this study also investigates the antioxidant activity of the extracts using DPPH assay to evaluate their free radical-scavenging potential. Additionally, this study examines the impact of extraction solvents (methanol vs. ethanol) on metabolite composition, highlighting the crucial role of solvent polarity in the efficiency of bioactive compound isolation. Understanding solvent-dependent extraction efficiency may facilitate the optimization of methods for obtaining fungal-derived therapeutic agents. By integrating chemical profiling with biological activity assessments, this study provides a comprehensive evaluation of *M. giganteus* and its potential applications in fungal biotechnology.

## 2. Materials and Methods

### 2.1. Stock Cultures

The fungal material used in this study consisted of an *M. giganteus* isolate obtained from the collection of pure mycelial cultures maintained at the Department of Forest Protection, Institute of Forest Sciences, Warsaw University of Life Sciences. To establish pure cultures, a small fragment of fruiting bodies was aseptically transferred onto 2% malt extract agar (MEA; Carl Roth, Karlsruhe, Germany) under a laminar flow hood. The mycelial cultures were stored at 5 °C on agar slants in glass tubes sealed with a cotton wool plug. To ensure culture viability, periodic subculturing onto freshly prepared MEA medium was performed every two years. As a reference material, fruiting bodies growing on Fagus spp. were collected in the vicinity of Warsaw in 2023.

To ensure genetic consistency, the strain used for all in vitro experiments originated from the same fruiting body collected on *Fagus* spp., from which a tissue fragment was aseptically transferred to malt extract agar to establish a pure culture.

To maintain culture viability, subculturing was carried out every two years.

### 2.2. Solid Medium Cultures

In the initial phase of the study, stationary cultures were established on agar medium in Petri dishes. The medium composition included 5% glucose, 1% yeast extract, 1% casein hydrolysate, 0.03% KH_2_PO_4_, and distilled water, with the pH adjusted to 6.5. A mycelial fragment from the stock culture was transferred to 60 mm glass Petri dishes containing the prepared medium. The cultures were incubated at approximately 22 °C under alternating light and dark conditions (simulating the natural circadian cycle) for 18 days.

### 2.3. Liquid Medium Cultures with Rotary Shaking

In the next step, submerged liquid cultures were initiated. Mycelial fragments from stationary cultures were aseptically transferred to 300 mL Erlenmeyer flasks containing 100 mL of sterile liquid medium. The composition of the liquid culture medium was identical to that described for the solid medium cultures (Section 2.2). This standardized composition was used throughout all stages of the biotechnological process to ensure comparability of biomass development and metabolite production. The flasks were sealed with 0.014 mm thick laboratory-grade aluminum foil and parafilm to prevent contamination. The cultures were incubated under continuous agitation at 140 revolutions per minute (RPM) on a rotary shaker (ALTEL, Łódź, Poland) at approximately 22 °C under alternating light and dark conditions following the circadian cycle for 18 days.

The 18-day cultivation period was selected based on preliminary trials and literature data [8,12], which indicated stable biomass development and consistent metabolite profiles at this stage of mycelial growth.

### 2.4. Bioreactor Cultures

The final stage of the biotechnological process involved the establishment of aerated cultures in air-lift bioreactors [12]. These cultures were cultivated in 2 L SIMAX^®^ glass bioreactors (Sázava, Czech Republic) containing 1.8 L of sterile medium. Mycelium from three-week-old submerged cultures was transferred into the bioreactors. Continuous stirring was ensured by an aeration system supplying a constant flow of sterile air through 0.22 µm PVDF syringe filters (Millipore, Merck, Darmstadt, Germany). Simultaneously, CO_2_ removal was facilitated using a Laboport^®^ mini vacuum pump (KNF, Freiburg, Germany). The cultures were maintained at approximately 22 °C under a photoperiod for a 10-day growth cycle. After the growth cycle, the biomass was harvested, washed with redistilled water, frozen, and subsequently freeze-dried using a Labconco freeze dryer (Labconco Corporation, Kansas City, MO, USA).

### 2.5. Extraction

The dried biomass and fruiting bodies were ground and subjected to extraction. Samples weighing 2 g were placed in 100 mL flasks and extracted with 20 mL of methanol (HPLC-grade) using ultrasonic-assisted extraction in an ultrasonic bath (2 × 30 min, 22 ± 3 °C). To prevent temperature increase, the water was replaced before each extraction cycle. The samples were centrifuged for 15 min (15,000× *g*, 4 °C). The resulting extracts were filtered through sterile syringe filters 0.22 μm, (Millipore, Merck, Darmstadt, Germany). Ethanolic extracts (HPLC-grade) were prepared analogously. Samples for each experimental group were prepared in triplicate. The extracts were stored at −4 °C for up to one week and used for all DAD-HPLC analyses.

### 2.6. HPLC Analysis of Bioactive Compounds

The quantification of lovastatin, ergothioneine, and indole compounds was performed using a high-performance liquid chromatography (HPLC) system (Merck Hitachi, Tokyo, Japan) including a diode array detector—DAD (L-7455), column oven (L-2350), pump (L-7100), autosampler (L-2200), degasser (VWR7614), and an RP-18 column (Purospher^®^, 4 × 250 mm, 5 µm). Lovastatin quantification followed the method of [13], using acetonitrile and 0.1% phosphoric acid (60:40) as the mobile phase, with a flow rate of 1 mL/min and detection at 238 nm. Ergothioneine determination, based on [14], employed a gradient elution with solvent A (water–methanol, 99:1, pH 5.0, adjusted with boric acid) and solvent B (HPLC-grade methanol). The flow rate was 0.5 mL/min, with detection at 257 nm. Indole compound quantification was adapted from [15] with in-house modifications [16], using methanol–water–0.1 M ammonium acetate (15:14:1) as the mobile phase, with a flow rate of 1 mL/min and detection at 275 nm. L-phenylalanine, syringic acid, and sterols were analyzed using an HPLC system (Merck Hitachi, Tokyo, Japan) equipped with an L-2455 DAD detector, column oven (L-2350), pump (L-2130), autosampler (L-2200), and an RP-18 column (LiChrosfer, 4 × 250 mm, 5 µm). L-phenylalanine and syringic acid were determined via gradient elution according to [17] with in-house modifications [18], using solvent A (methanol–0.5% acetic acid, 1:4) and solvent B (HPLC-grade methanol) with a flow rate of 1 mL/min and detection at 254 nm. Sterol quantification followed the method of [19] with in-house modifications [20], using solvent A (methanol–water, 80:20) and solvent B (methanol–dichloromethane, 75:25) under a gradient program, with a flow rate of 1 mL/min and detection at 280 nm. Quantification was based on calibration curves assuming a linear correlation between peak area and concentration.

### 2.7. Cell Culture and Conditions

The experiment was conducted on human cancer cell lines, representing the gastrointestinal panel: DLD-1 colon adenocarcinoma (ATCC CCL-221), HCT116 colon carcinoma (ATCC CCL-247), HT29 colorectal adenocarcinoma (ATCC HTB-38), and hepatocellular carcinoma HepG2 (ATCC HB-8065), and non-cancerous human colon epithelial cell line CCD 841 CoN (CRL-1790). Cells were grown at 37 °C in a 5% CO_2_ atmosphere, with relative humidity, in culture medium McCoy’s (HT29), Dulbecco’s Modified Eagle Medium (DMEM) high glucose (HCT116 and DLD-1), or minimal essential medium (MEM)—(HepG2, CCD 841 CoN), supplemented with 10% fetal bovine serum (FBS) and 1% antibiotics solution (10,000 U penicillin and 10 mg streptomycin/mL). All culture media and supplements were obtained from Merck (Darmstadt, Germany). The tested extracts were dissolved in methanol or ethanol to obtain stock solutions of 10 mg/mL, and further diluted with culture medium to the working concentrations. The influence of methanol and ethanol on cell viability was examined, and no toxic effect was noted.

### 2.8. Cell Viability Assay

Before the experiment, cells were seeded onto 96-well plates (1.5 × 10^4^ cells/well) for 24 h. Then, the tested samples were added (0 to 200 µg/mL) and incubated for 48 h. Cell viability was measured with MTT assay (3-(4,5-dimethylthiazol-2-yl)-2,5-diphenyltetrazolium bromide) (Merck, Darmstadt, Germany), as described previously [21]. Doxorubicin was used as a reference drug control. The absorbance was measured at 570 nm using a Biotek Synergy microplate reader (BioTek Instruments Inc., Winooski, VT, USA). Cell viability was expressed as a % of control (untreated cells). Each experiment was performed in triplicate.

### 2.9. DPPH Radical Scavenging Activity

The antioxidant activity of methanolic and ethanolic extracts from the mycelia and fruiting bodies of MG was assessed using the 2,2-diphenyl-1-picrylhydrazyl (DPPH) assay, following a modified Prieto protocol [22]. A 100 μL aliquot of 0.2 mM DPPH solution (prepared in methanol or ethanol, depending on the extract type) was mixed with 100 μL of the sample of different extract concentration in a 96-well flat-bottom microplate. The plate was incubated in the dark at room temperature for 50 min, the time required for the reaction to reach equilibrium according to preliminary experiments Trolox^®^ (Thermo Scientific Chemicals, Waltham, MA, USA) was used as a positive control, and methanol or ethanol were used as negative control. Absorbance was then measured at 517 nm using a Synergy H1 Multi-Mode Microplate Reader (BioTek^®^ Instruments), and the DPPH removal was calculated using Equation (1).

(1) $$DPPH\;scavenging\;activity=\left(\frac{A_{DPPH}-A_{Sample}}{A_{DPPH}}\right)\times 100\;[\%]$$

where

A_DPPH_—absorbance of DPPH 0.1 mM solution;
A_Sample_—absorbance of the test sample.

Change in the absorbance value was plotted against sample extract concentration, and a linear regression curve was established in order to calculate the EC_50_ (Effective Concentration), which refers to the concentration of an antioxidant (e.g., plant extract or pure compound) required to scavenge 50% of the DPPH free radicals.

The antioxidant activity of the test sample, determined using the DPPH assay, was also determined relative to that of Trolox^®^, a water-soluble vitamin E analog. Values were reported as Trolox equivalent antioxidant capacity (TEAC), an index of antioxidant activity of the sample relative to Trolox^®^. TEAC values were computed from Equation (2).

(2) $$TEAC=\frac{{EC}_{50\;TROLOX}}{{EC}_{50\;Sample}}\times 10^{6}\;[µg\;Trolox\;per\;g\;DW\;of\;sample]$$

Extract concentrations ranging from 10 to 100 mg/mL were evaluated in the assay. The exact linear ranges used for EC_50_ calculation varied depending on extract type and are specified in the Results section (Section 3.7). All measurements were performed in triplicate.

### 2.10. Statistical Analysis

All experiments were conducted in triplicate, and results are presented as mean ± standard deviation (SD). Data were analyzed using one-way ANOVA followed by Tukey’s post hoc test to assess differences between groups. Statistical calculations were performed in STATISTICA v.14 (TIBCO Software Inc., Palo Alto, CA, USA). A *p*-value ≤ 0.05 was considered statistically significant. Due to default output settings of the software, exact *p*-values were not exported; therefore, significance is reported as *p* ≤ 0.05 or *p* > 0.05, as appropriate.

## 3. Results and Discussion

### 3.1. Mycelial Growth

**Figure 1:**
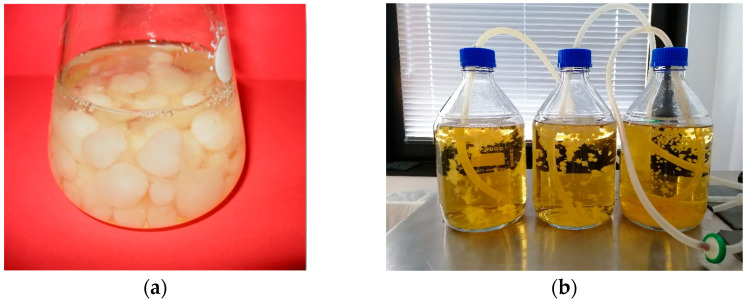
Mycelial cultures of *Meripilus giganteus*: (**a**) submerged cultures grown in liquid medium; (**b**) aerated cultures in air-lift bioreactors.

After 10 days of cultivation, mycelial cultures grown in liquid medium in air-lift bioreactors were evaluated. Biomass growth and morphology were assessed. Throughout the experiment, homogeneous, contamination-free mycelial cultures were obtained in all series. Regular microscopic observations confirmed the absence of bacterial contamination and other fungal strains. The appearance of the tested cultures, whether grown in submerged conditions or in air-lift bioreactors, exhibited similarities (Figure 1). At the end of the 10-day cultivation period, the biomass was harvested, yielding 4.5 g of dry weight (DW) per liter of medium.

### 3.2. Biological Active Compounds’ Contents and Extraction Efficiency

**Figure 2:**
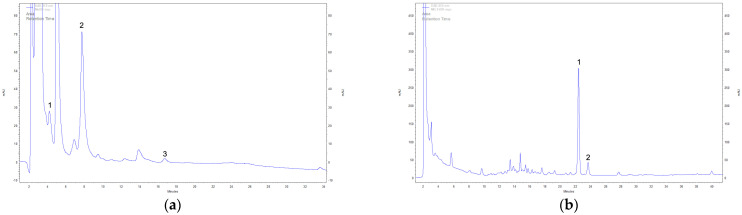
Example DAD-HPLC chromatogram of extracts cultivated under in vitro conditions. Identified compounds in methanolic extracts of mycelium: (**a**) 1—L-phenylalanine, 2—L-tryptophan, 3—syringic acid; identified compounds in ethanolic extracts of mycelium: (**b**) 1—ergosterol, 2—tocopherol.

Using the DAD-HPLC method on methanolic and ethanolic extracts, the presence of 11 secondary metabolites was identified (Table 1 and Figure 2). The analysis of sterols, specifically ergosterol and tocopherol content in extracts, demonstrated significant variations depending on fungal growth form (fruiting bodies vs. mycelium) and the solvent used for extraction. This study provides valuable insights into the metabolic differences between fruiting bodies and mycelium, as well as the solvent-dependent extraction efficiencies of these bioactive compounds.

Ergosterol, a key sterol component of fungal cell membranes, varied significantly across extracts [23]. The methanolic extract from fruiting bodies exhibited the highest ergosterol content (147.85 mg/100 g DW) (Figure S1), whereas the corresponding mycelium extract contained a significantly lower amount (89.56 mg/100 g DW). Conversely, when ethanol was used as the extraction solvent, the trend was reversed: mycelial biomass yielded the highest ergosterol content (178.67 mg/100 g DW), whereas fruiting bodies contained the lowest amount (57.21 mg/100 g DW). These differences suggest that ergosterol accumulation is influenced by fungal developmental stage and cell-wall structure, with fruiting bodies favoring methanol-based extraction, while ethanol more efficiently extracts ergosterol from mycelium.

In turn, tocopherol exhibited a different accumulation pattern. Mycelium consistently showed higher tocopherol concentrations compared to fruiting bodies, regardless of the extraction solvent. In methanolic extracts, the tocopherol content was 54.23 ± 6.53 mg/100 g DW for mycelium and 40.59 ± 0.23 mg/100 g DW for fruiting bodies. Similarly, in ethanolic extracts, the values were 69.00 ± 0.21 mg/100 g DW for mycelium and 25.91 ± 5.02 mg/100 g DW for fruiting bodies. These data confirm that mycelium consistently accumulates more tocopherol than fruiting bodies, regardless of the solvent used. These findings indicate that *M. giganteus* mycelium may serve as a superior source of tocopherol, potentially influenced by growth conditions in submerged culture systems, such as controlled nutrient availability and oxygen diffusion.

Lovastatin, a secondary metabolite with cholesterol-lowering properties, exhibited significant differences depending on fungal growth form and the solvent used. The highest lovastatin yield was observed in the methanolic extract of fruiting bodies (12.46 mg/100 g DW), followed by ethanol-extracted fruiting bodies (5.02 mg/100 g DW) (Figure S2).

In contrast, the methanolic extract of mycelium contained only 3.42 mg/100 g DW (Figure S3), while ethanol extraction yielded the lowest concentration (2.27 mg/100 g DW). These results indicate that fruiting bodies accumulate higher amounts of lovastatin than mycelium, suggesting that submerged cultures may not provide optimal conditions for lovastatin biosynthesis. Additionally, methanol was found to be a more effective solvent for extracting lovastatin than ethanol, yielding significantly higher concentrations in both fruiting bodies and mycelium. This highlights the need for further optimization of extraction protocols, including potential metabolic engineering approaches to enhance lovastatin biosynthesis in submerged cultures.

Ergothioneine, a potent antioxidant with cytoprotective properties, was found to accumulate predominantly in mycelium. The highest ergothioneine content was detected in the methanolic extract of mycelium (44.31 mg/100 g DW), which was significantly higher than that in fruiting bodies (21.17 mg/100 g DW) (Figure S4).

A similar trend was observed in ethanol extracts, where mycelium contained 17.63 mg/100 g DW (Figure S5), while fruiting bodies yielded only 8.50 mg/100 g DW. These results suggest that submerged mycelial cultures may be a more efficient and scalable source of ergothioneine than fruiting bodies. Furthermore, methanol was the more effective solvent for ergothioneine extraction, likely due to its ability to disrupt fungal cell walls and solubilize intracellular components.

L-phenylalanine, an aromatic amino acid involved in neurotransmitter biosynthesis, was predominantly found in fruiting bodies, with the highest concentration detected in methanolic extracts (4.95 mg/100 g DW). In contrast, mycelium contained significantly lower amounts (1.89 mg/100 g DW), suggesting that L-phenylalanine biosynthesis may be more active in fruiting bodies.

Syringic acid, a phenolic compound with antioxidant properties, was detected exclusively in mycelium, with the highest concentration found in ethanol extracts (0.16 mg/100 g DW), followed by methanol extracts (0.26 mg/100 g DW).

The analysis of indole derivatives revealed significant differences in L-tryptophan, serotonin, and 5-hydroxytryptophan (5-HTP) accumulation between fruiting bodies and mycelium. The highest L-tryptophan concentration was detected in the ethanol extract of mycelium (57.13 mg/100 g DW), followed by ethanol-extracted fruiting bodies (38.16 mg/100 g DW) (Figure S6). Methanol extraction yielded lower concentrations, with 28.37 mg/100 g DW in mycelium and only 7.05 mg/100 g DW in fruiting bodies. These results indicate that mycelium is a richer source of L-tryptophan than fruiting bodies and that ethanol is the preferred solvent for its extraction.

Serotonin content was significantly higher in mycelium compared to fruiting bodies. In methanol extracts, the levels were 17.22 ± 6.27 mg/100 g DW for mycelium and 1.82 ± 0.02 mg/100 g DW for fruiting bodies. In ethanol extracts, the serotonin content reached 3.53 ± 0.30 mg/100 g DW in mycelium and 2.92 ± 0.04 mg/100 g DW in fruiting bodies. These results confirm that mycelium is a richer source of serotonin than fruiting bodies and that methanol is a slightly more effective solvent for serotonin extraction.

5-Hydroxytryptophan (5-HTP), a precursor to serotonin, was detected exclusively in mycelium, albeit at low concentrations (0.41 mg/100 g DW in methanol extracts and 0.82 mg/100 g DW in ethanol extracts), while fruiting bodies contained none. This suggests that mycelial cultures may provide a more favorable environment for the biosynthesis of serotonin precursors.

### 3.3. Biotechnological and Pharmaceutical Implications

The quantitative results obtained using DAD-HPLC analysis revealed that mycelial biomass contained significantly higher concentrations of selected compounds compared to fruiting bodies. Notably, ergothioneine reached 44.31 mg/100 g DW, tocopherol up to 69.00 mg/100 g DW, and L-tryptophan up to 57.13 mg/100 g DW. In contrast, fruiting bodies showed higher levels of lovastatin (up to 12.46 mg/100 g DW) and L-phenylalanine (4.95 mg/100 g DW). These data indicate that mycelium and fruiting bodies differ in their metabolite profiles, suggesting distinct applications.

The observed variations in bioactive compound accumulation between fruiting bodies and mycelium underscore the importance of selecting optimal fungal growth conditions and extraction methods to maximize metabolite yields [24]. The significantly higher levels of ergosterol, tocopherol, ergothioneine, and L-tryptophan in mycelium suggest that submerged mycelium could be utilized for the large-scale production of these valuable compounds. While fruiting bodies exhibited higher levels of lovastatin and L-phenylalanine, their limited seasonal availability presents a challenge for industrial applications. The lower concentrations of lovastatin in mycelium suggest that additional optimization strategies, such as precursor supplementation or genetic modifications, may be necessary to enhance its biosynthesis. Solvent selection played a crucial role in extraction efficiency, with methanol proving to be more effective for ergosterol, serotonin, and ergothioneine, whereas ethanol was more efficient for L-tryptophan and syringic acid.

These findings highlight the potential of *M. giganteus* as a biotechnologically valuable species for pharmaceutical, nutraceutical, and functional food applications, warranting further research into optimizing cultivation and extraction strategies [24].

### 3.4. Therapeutic Properties of Bioactive Compounds

The analysis of extracts revealed significant levels of ergosterol and tocopherol, compounds with well-documented health benefits. Ergosterol serves as a precursor to vitamin D₂, which is essential for calcium homeostasis and bone health [25]. The high ergosterol content detected in *M. giganteus* suggests its potential as a natural source for vitamin D_2_ synthesis [26]. In fungal cells, ergosterol biosynthesis is tightly regulated by multiple transcription factors, including Upc2 and Ecm22, which respond to sterol availability. This regulation ensures membrane integrity and adaptation to environmental stressors, while also making ergosterol a primary target of antifungal agents such as azoles and polyenes [23]. Additionally, ergosterol can interfere with cholesterol metabolism and has been suggested to exhibit cytotoxic effects by disrupting lipid rafts in cancer cells, thereby affecting cell signaling pathways involved in apoptosis and proliferation [27].

Tocopherol, commonly known as vitamin E, is a potent antioxidant that protects cellular membranes from oxidative damage [28]. The substantial tocopherol concentrations in mycelium indicate that *M. giganteus* could serve as a valuable natural source of antioxidants, potentially useful in preventing oxidative stress-related diseases such as cardiovascular disorders and neurodegenerative conditions [29,30]. Tocopherol modulates cellular signaling pathways. It neutralizes lipid radicals, preventing the propagation of lipid peroxidation within phospholipid membranes. This action maintains membrane integrity and protects against oxidative damage in tissues, including the cardiovascular and nervous systems [29]. Additionally, tocopherol interacts with nuclear receptors such as peroxisome proliferator-activated receptor gamma (PPARγ), which plays a role in modulating inflammation and lipid metabolism. It has also been reported to inhibit nuclear factor kappa B (NF-κB) activation, a key mediator of inflammation, reducing NF-κB signaling and exerting anti-inflammatory effects [31].

Lovastatin, a secondary metabolite with cholesterol-lowering properties, was detected in varying concentrations across extracts. As a competitive inhibitor of HMG-CoA reductase, lovastatin effectively reduces low-density lipoprotein (LDL) cholesterol levels, thereby lowering the risk of atherosclerosis and cardiovascular diseases [25,32]. The higher concentrations in fruiting bodies suggest that *M. giganteus* may have applications in functional foods or nutraceutical formulations targeting cholesterol management. However, the relatively lower levels in mycelium indicate the need for metabolic optimization strategies, such as precursor supplementation or biotechnological approaches, to enhance lovastatin biosynthesis in submerged cultures. Statins, such as lovastatin, are well-established inhibitors of 3-hydroxy-3-methylglutaryl-CoA (HMG-CoA) reductase, the enzyme responsible for cholesterol biosynthesis. Their therapeutic benefits extend beyond lipid-lowering effects. By inhibiting HMG-CoA reductase, statins reduce the production of mevalonate, a precursor for cholesterol and other isoprenoids. This inhibition not only lowers cholesterol levels but also affects cell signaling pathways associated with cell proliferation and immune function [32]. Statins enhance eNOS activity, increasing nitric oxide (NO) production. This action promotes vasodilation, improves endothelial function, and provides cardiovascular protection [33]. Statins have also been reported to induce apoptosis in cancer cells via modulation of the Ras/MAPK and PI3K/Akt pathways, contributing to their emerging role in cancer chemoprevention and therapy [34].

Ergothioneine is a naturally occurring thiol compound with strong antioxidant and cytoprotective properties. It plays a crucial role in cellular defense against oxidative stress, exhibiting potential benefits in neuroprotection and anti-inflammatory responses [35]. The higher ergothioneine content in mycelium compared to fruiting bodies suggests that submerged cultures could serve as an efficient and scalable source of this compound.

Given its emerging role in preventing chronic inflammatory diseases and neurodegenerative disorders, *M. giganteus* mycelium may be considered for future pharmacological and nutraceutical applications. Ergothioneine plays a crucial role in cellular defense mechanisms. It is actively transported into cells via the organic cation transporter OCTN1 (SLC22A4), indicating its physiological importance in oxidative stress regulation. Ergothioneine regulates oxidative stress by modulating the nuclear factor erythroid 2-related factor 2 (NRF2) pathway. It interacts with Kelch-like ECH-associated protein 1 (KEAP1), leading to NRF2 stabilization and nuclear translocation. NRF2 then binds to antioxidant response elements (ARE) in the DNA, upregulating genes responsible for detoxification and oxidative stress defense, such as heme oxygenase-1 (HO-1) and glutathione S-transferases [36].

Sirtuin-mediated cellular protection is another mechanism influenced by ergothioneine. It has been shown to enhance the activity of sirtuins (SIRT1 and SIRT6), NAD^+^-dependent deacetylases that regulate cellular homeostasis, mitochondrial function, and genomic stability. Increased sirtuin activity has been linked to anti-aging and neuroprotective effects [36].

L-phenylalanine, an essential aromatic amino acid, serves as a precursor for neurotransmitter biosynthesis, including dopamine and norepinephrine [37]. Its presence in fruiting bodies suggests a potential role of *M. giganteus* in supporting cognitive function and mental health. It influences neural function and protein metabolism. L-phenylalanine metabolism is closely linked to the phenylalanine hydroxylase (PAH) pathway, and its dysregulation has been associated with metabolic disorders and neurological diseases [37].

Meanwhile, syringic acid, a phenolic compound with antioxidant and anti-inflammatory properties, was detected exclusively in mycelium. Syringic acid has been shown to exhibit protective effects against oxidative damage and may have applications in anti-aging and anti-inflammatory therapies [38,39]. It has been reported to modulate enzyme activity involved in detoxification processes and interact with signaling pathways associated with inflammatory responses, such as the NF-κB and MAPK pathways. Studies suggest that syringic acid can scavenge free radicals, inhibit pro-inflammatory cytokine production, and enhance the activity of endogenous antioxidant enzymes [39].

These findings highlight the differential metabolic profiles of *M. giganteus* fruiting bodies and mycelium, indicating their potential in distinct therapeutic areas. The quantification of L-tryptophan and its derivatives in *M. giganteus* extracts suggests potential neuropharmacological applications. L-tryptophan, a precursor of serotonin, plays a vital role in mood regulation, sleep, and appetite control [11,40,41]. The significantly higher L-tryptophan levels in mycelium indicate that submerged cultures could be optimized as a natural source of this essential amino acid. Moreover, serotonin, a key neurotransmitter associated with mental well-being, was found in greater abundance in mycelium, particularly in methanolic extracts. The detection of 5-hydroxytryptophan (5-HTP), a direct precursor to serotonin, exclusively in mycelium further supports the potential of *M. giganteus* mycelium as a source of neuroactive compounds with applications in mood disorders and cognitive enhancement.

### 3.5. Biotechnological Relevance of Quantified Metabolites

The differences in metabolite profiles observed between *M. giganteus* fruiting bodies and mycelium point to promising applications of in vitro culture in the sustainable production of specific bioactive compounds. Based on the comparative profiles, mycelial cultures demonstrated potential for the scalable production of selected compounds, particularly serotonin, ergothioneine, and L-tryptophan. In contrast, fruiting bodies proved to be a superior source of lovastatin and L-phenylalanine. These findings support the application of different fungal sources depending on the desired compound, and they provide a foundation for further biotechnological development. It should be noted, however, that our current methodology does not account for potential genetic drift during long-term mycelial culture maintenance. This limitation will be addressed in future work focusing on strain stability and genetic fidelity during prolonged subculturing.

In comparison with other well-studied medicinal fungi, the mycelium of *M. giganteus* shows competitive or even superior levels of several metabolites. For example, the ergothioneine content in methanolic mycelial extracts (44.31 mg/100 g DW) exceeds that typically reported for *Pleurotus ostreatus* and *Lentinula edodes* (10–30 mg/100 g DW) [42]. Similarly, tocopherol concentrations (up to 69.00 mg/100 g DW in ethanolic extracts) surpass those found in *Ganoderma lucidum* and *Inonotus obliquus* [43]. The relatively high levels of L-tryptophan (57.13 mg/100 g DW) in ethanolic mycelial extracts further indicate neuroactive potential. Conversely, lovastatin levels in *M. giganteus* fruiting bodies (12.46 mg/100 g DW) are lower than those in *Aspergillus terreus* under optimized conditions, where they may exceed 168.5 mg/100 g DW [13].

From a pharmaceutical perspective, ergothioneine is under investigation for its cytoprotective and anti-aging properties, tocopherol remains a key antioxidant in both dermatology and nutrition, and L-tryptophan plays a role in serotonin synthesis and mood regulation. These metabolites, when sourced from in vitro fungal cultures, offer scalability and environmental sustainability. Although *M. giganteus* may not be the most efficient producer of all target compounds, it offers substantial biotechnological potential—particularly for antioxidant and neuroprotective metabolite production.

### 3.6. Cytotoxicity

The cytotoxic activity of methanol and ethanol extracts from *Meripilus giganteus* fruiting bodies and mycelium was assessed using the MTT assay. Only moderate effects were observed at the highest tested concentration (200 µg/mL), and no statistically significant differences were noted between extracts derived from different fungal growth forms. Among the cancer cell lines tested, HT29 colon adenocarcinoma cells showed the highest sensitivity, especially to ethanol extracts, while HCT116 cells responded more strongly to methanol-based extracts. HepG2 hepatocellular carcinoma cells were the most resistant.

**Figure 3:**
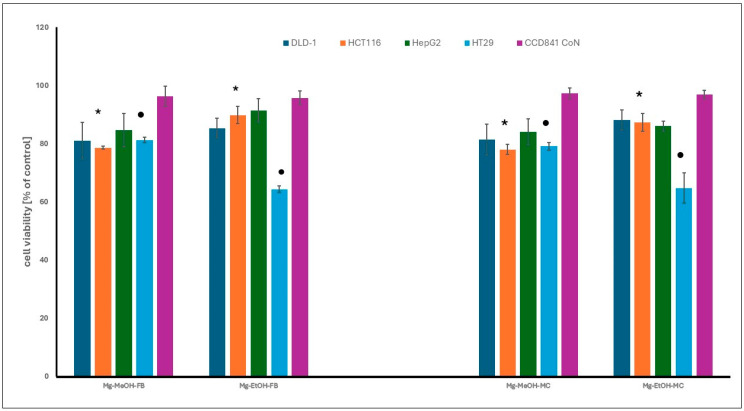
The cytotoxicity of fruiting bodies (FB) and mycelium (MYC) extracts of *M. giganteus* against gastrointestinal cancer and normal cell lines, at the highest tested concentration (200 µg/mL) after 48 h. The results are expressed as a percentage of viable cells relative to the control, untreated cells. The differences observed were statistically significant (*p* < 0.05). The asterisk (*) indicates significant differences between ethanol and methanol extracts for the HCT 116 cell line. The dot (•) denotes significant differences between ethanol and methanol extracts for the HT29 cell line. The explanation of extracts symbols and cell lines are presented in the Section 2.

None of the extracts showed cytotoxicity against non-neoplastic colon epithelial cells (CCD 841 CoN), suggesting a favorable selectivity profile (Figure 3). These results indicate that *M. giganteus* extracts may possess selective antiproliferative properties, particularly against colon cancer cells. However, the tested extracts were complex mixtures obtained without compound purification or standardization.

While the mechanisms underlying the observed cytotoxicity remain to be elucidated, previous studies have reported that certain fungal metabolites, such as ergothioneine, phenolic acids, and indole derivatives, may exert their effects through pathways involving oxidative stress or apoptosis [44,45]. In the current study, no mechanistic assays were conducted; therefore, such interpretations remain speculative and warrant further investigation using targeted molecular approaches.

The cytotoxic effects of *M. giganteus* extracts on colorectal adenocarcinoma cells are likely mediated through the induction of apoptosis via the modulation of pro- and anti-apoptotic proteins, the enforcement of cell-cycle arrest at critical checkpoints, and the elevation of ROS levels leading to oxidative stress [46,47].

Understanding these molecular mechanisms provides valuable insights into the potential therapeutic applications of *M. giganteus* in cancer treatment.

### 3.7. DPPH Radical Scavenging Activity

The DPPH radical scavenging activity of methanolic and ethanolic extracts of *M. giganteus* is presented in Figure S7. A linear relationship between DPPH radical scavenging and extract concentration was observed within the following ranges: 20.0–90.0 mg/mL for Mg-MeOH-MC, 10.0–40.0 mg/mL for Mg-MeOH-FB, 10.0–100.0 mg/mL for Mg-EtOH-MC, and 20.0–100.0 mg/mL for Mg-EtOH-FB. The obtained linear regression equation was used to calculate EC_50_ values, which are presented in Table 2.

The linear relationship between extract concentration and DPPH radical scavenging activity, which served as the basis for EC_50_ calculation, is presented in the Supplementary Materials (Figure S8).

The EC_50_ values for methanolic and ethanolic TROLOX^®^ solutions were determined using the linear regression equations derived from the data presented in Figure S8. The linear range was observed for Trolox^®^ concentrations between 0.001 and 0.008 mg/mL. The EC_50_ values for methanolic and ethanolic TROLOX^®^ solutions were 0.00495 and 0.00589 mg/mL, respectively.

Methanol extracts possessed higher antioxidant capacity, as manifested in terms of DPPH assay results and EC_50_ values (Table 2). The lower the EC_50_ value, the lower the concentration of antioxidants required to reduce the initial DPPH radical concentration by 50%. Mg-MeOH-FB exhibited the strongest DPPH radical scavenging activity among all the extracts evaluated, while Mg-EtOH-FB showed the lowest DPPH radical reduction activity.

The DPPH assay relies on the ability of antioxidants to neutralize free radicals by donating either a proton or an electron. In the case of *M. giganteus*, potential mechanisms include hydrogen-atom transfer (HAT), where antioxidants in the extracts donate a hydrogen atom, leading to the reduction in the DPPH˙ radical to its inactive form, DPPH-H, and single-electron transfer (SET), in which antioxidants donate a single electron, stabilizing the DPPH˙ radical. Differences in EC_50_ values among the extracts may indicate that distinct fractions are dominated by compounds with varying mechanisms of action.

The antioxidant activity observed in the tested extracts can be attributed primarily to the presence of phenolic compounds (e.g., syringic acid), indole derivatives (e.g., L-tryptophan, serotonin), tocopherols, and sulfur-containing antioxidants such as ergothioneine, all of which have been reported to effectively scavenge free radicals through hydrogen-atom transfer (HAT) or single-electron transfer (SET) mechanisms. EC_50_ values have been determined in various studies involving different mushroom species. The EC_50_ values obtained in this study (e.g., 28.18 mg/mL for methanolic fruiting body extract and 48.05 mg/mL for methanolic mycelial extract) suggest moderate antioxidant potential. For comparison, EC_50_ values reported for *Inonotus obliquus* range from 12 to 30 mg/mL depending on extraction method [48], while *Ganoderma lucidum* extracts typically fall within the 25–50 mg/mL range [49], and *Laetiporus sulphureus* methanolic extracts have demonstrated EC_50_ values around 35–45 mg/mL [6]. However, it is important to note that directly comparing EC_50_ values from different studies can lead to incorrect conclusions, as these values are highly dependent on the specific conditions of the assay protocols used [50].

### 3.8. Cytotoxicity Data Interpretation

The observed moderate cytotoxicity of *M. giganteus* extracts toward cancer cell lines, especially HT29 and HCT116, may be related to differences in extract composition or cancer cell sensitivity. The lack of toxicity toward normal colon epithelial cells (CCD 841 CoN) is encouraging but may be attributed, at least in part, to the slower proliferation rate of these cells [51].

While fungal secondary metabolites such as phenolic acids, indoles, and statins are known to modulate cell viability through apoptosis induction, oxidative stress, or cell-cycle arrest, the present study was limited to a viability assay and did not include molecular analyses. Therefore, any discussion of underlying mechanisms remains speculative. Further investigations are required to confirm whether pathways such as ROS-mediated apoptosis or caspase activation are involved in the bioactivity of the extracts [46,47].

## 4. Conclusions

As the demand for natural bioactive compounds grows, understanding the potential of mycelium as an alternative source of fungal metabolites may open new avenues for the sustainable and controlled production of therapeutic agents. Given the urgent need for novel antioxidant and anticancer strategies in gastrointestinal cancer prevention and treatment, further exploration of *M. giganteus* could contribute to the development of new supportive therapies targeting oxidative stress and inflammation-driven oncogenesis. In conclusion, *M. giganteus* represents a promising candidate for further research in the field of gastrointestinal cancer chemoprevention. However, more detailed studies are needed to fully elucidate its therapeutic potential. To our knowledge, this is the first comparative analysis of in vitro-cultivated mycelium and fruiting bodies of *M. giganteus* with respect to chemical composition and biological activity.

## Figures and Tables

**Table 1:** The contents of selected organic compounds in *Meripilus giganteus* extracts from fruiting bodies and mycelium expressed as mean ± standard deviation (mg/100 g DW).

Analyzed Compounds		Mg-MeOH-FB	Mg-MeOH-MC	Mg-EtOH-FB	Mg-EtOH-MC
Sterols	Ergosterol	147.85 ± 0.54 ^b^	89.56 ± 2.58 ^c^	57.21 ± 1.71 ^d^	178.67 ± 0.66 ^a^
	Tocopherol	40.59 ± 0.23 ^c^	54.23 ± 6.53 ^b^	25.91 ± 5.02 ^d^	69.00 ± 0.21 ^a^
	Ergosterol peroxide	*	*	*	*
Indole compounds	L-Tryptophan	7.05 ± 0.12 ^a^	28.37 ± 1.04 ^c^	38.16 ± 0.95 ^b^	57.13 ± 0.25 ^a^
	Serotonin	1.82 ± 0.02 ^b^	17.22 ± 6.27 ^a^	2.92 ± 0.04 ^b^	3.53 ± 0.30 ^b^
	5-Hydroxy-tryptophan	nd	0.41 ± 0.03 ^b^	nd	0.82 ± 0.01 ^a^
Proteinogenic amino acid	L-phenylalanine	4.95 ± 0.35 ^a^	1.89 ± 0.05 ^b^	2.02 ± 0.49 ^b^	1.09 ± 0.02 ^c^
Phenolic acids	Syringic acid	nd	0.26 ± 0.03 ^a^	nd	0.16 ± 0.02 ^b^
Statins	Lovastatin	12.46 ± 0.08 ^a^	3.42 ± 0.01 ^c^	5.02 ± 0.01 ^b^	2.27 ± 0.08 ^d^
Sulfur-containing antioxidants	Ergothioneine	21.17 ± 2.98 ^b^	44.31 ± 7.73 ^a^	8.50 ± 1.10 ^c^	17.63 ± 3.56 ^b,c^

Mg-MeOH-FB—*Meripilus giganteus* methanolic extract of fruiting body; Mg-MeOH-MC—*Meripilus giganteus* methanolic extract of mycelium; Mg-EtOH-FB—*Meripilus giganteus* ethanolic extract of fruiting body; Mg-EtOH-MC—*Meripilus giganteus* ethanolic extract of mycelium; *—trace amounts; nd—not detected. Data are presented as mean ± standard deviation. The Tukey test was used to identify differences between groups of metabolites in columns when comparing the type of elicitor and its concentrations with the control, separately for each species. Different letters (^a^, ^b^, ^c^ and ^d^) indicate statistically significant differences in content (*p* < 0.05).

**Table 2:** EC_50_ values of the tested extracts and their corresponding Trolox equivalent antioxidant capacity (TEAC). EC_50_ values were calculated based on the regression equations presented in Supplementary Figure S7.

Extract	EC_50_ [mg/mL]	TEAC [µg of TE/g of Sample]
	Methanol extracts	
Mg-MC	48.05	103.1
Mg-FB	28.18	175.66
	Ethanol extracts	
Mg-MC	63.43	92.86
Mg-FB	114.39	51.15

Mg-FB—*Meripilus giganteus* fruiting body, Mg-MC—*Meripilus giganteus* mycelium.

## Data Availability

The original contributions presented in this study are included in the article/Supplementary Materials. Further inquiries can be directed to the corresponding author.

## Supplementary Materials

The supporting information can be downloaded at: https://www.mdpi.com/article/10.3390/cimb47050302/s1.

## Abbreviations

The following abbreviations are used in this manuscript:

ABTS	2,2′-azino-bis(3-ethylbenzothiazoline-6-sulfonic acid)
CCD 841 CoN	Human normal colon epithelial cell line
DAD-HPLC	Diode-Array Detection High-Performance Liquid Chromatography
DLD-1	Human colorectal adenocarcinoma cell line
DMEM	Dulbecco’s Modified Eagle Medium
DPPH	2,2-diphenyl-1-picrylhydrazyl
DW	Dry Weight
FBS	Fetal Bovine Serum
HCT116	Human colorectal carcinoma cell line
HepG2	Human hepatocellular carcinoma cell line
HPLC	High-Performance Liquid Chromatography
HT29	Human colorectal adenocarcinoma cell line
LDL	Low-Density Lipoprotein
MEA	Malt Extract Agar
MEM	Minimum Essential Medium
MTT	3-(4,5-Dimethylthiazol-2-yl)-2,5-diphenyltetrazolium bromide assay
MC	Mycelial Culture
PVDF	Polyvinylidene Difluoride
RPM	Revolutions Per Minute
ROS	Reactive Oxygen Species
