# Supplementary material for: A Comparative Study on the Mycelium and Fruiting Body of Meripilus giganteus: Chemical Composition and Biological Activity
Source: Curr Issues Mol Biol. 2025 Apr 25;47(5):302. doi: 10.3390/cimb47050302 (PMC12110640; doi:10.3390/cimb47050302)
Supplement: Supplementary file 1 [file cimb-47-00302-s001.zip › cimb-3550052-supplementary.pdf]

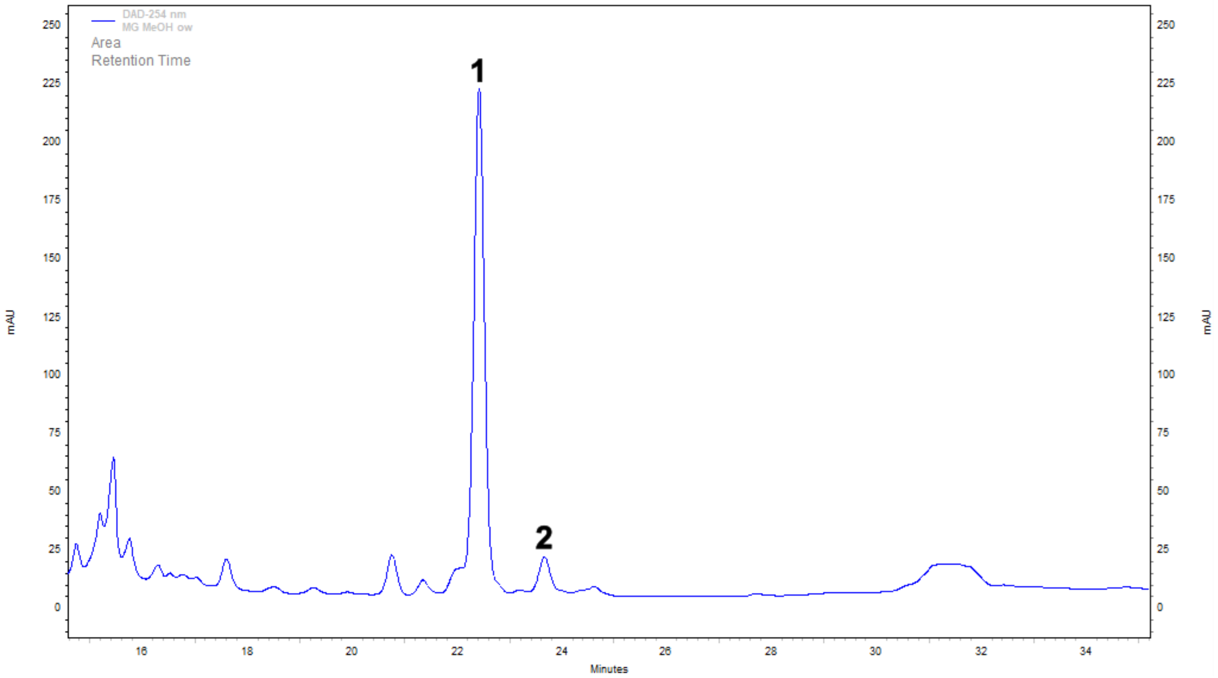

Figure S-1. Example DAD-HPLC chromatogram of a methanolic extract from the fruiting body of *Merililus giganteus*, showing peaks for (1) ergosterol and (2) tocopherol.

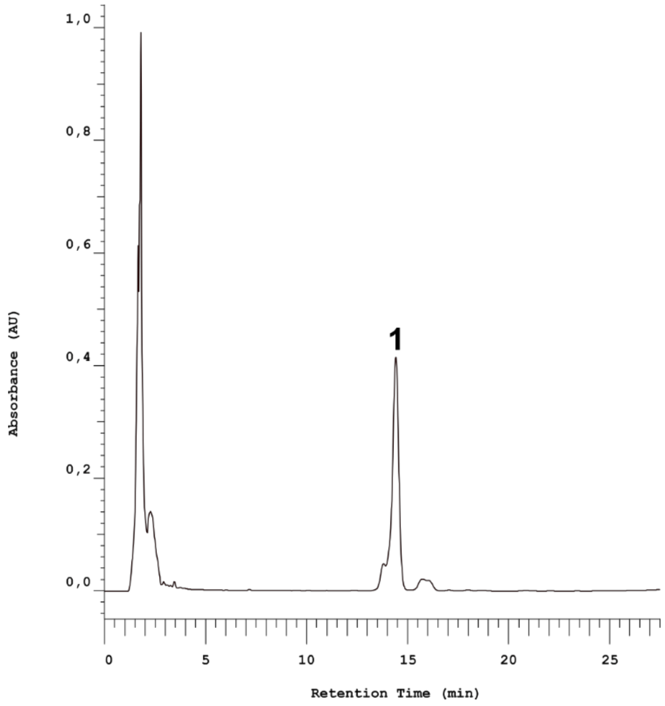

Figure S-2. Example DAD-HPLC chromatogram of an ethanolic extract from the fruiting body of *Meripilus giganteus*, showing the peak for (1) lovastatin.

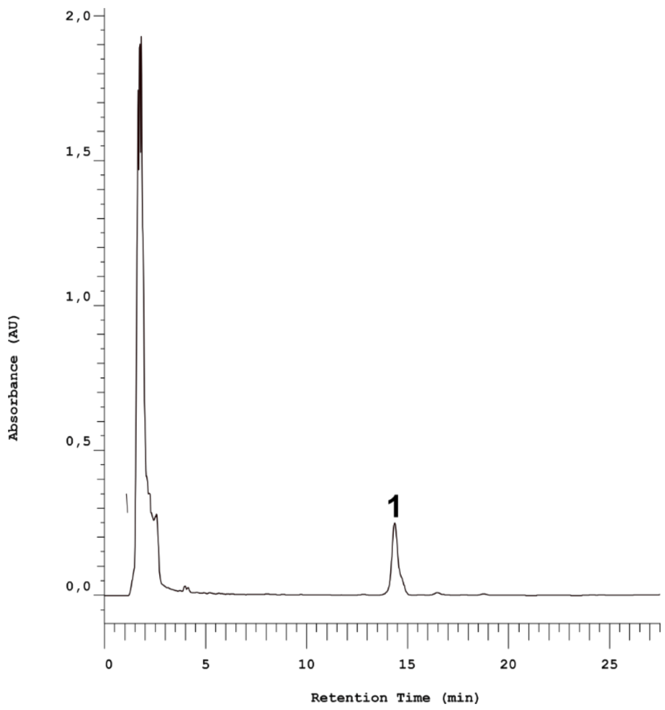

Figure S-3. Example DAD-HPLC chromatogram of a methanolic extract from *Meripilus giganteus* mycelium, showing the peak for (1) lovastatin.

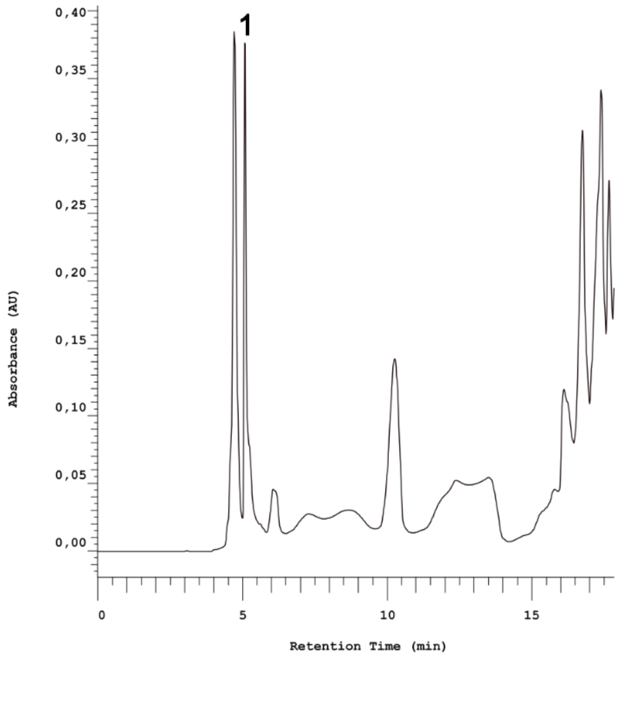

Figure S-4. Example DAD-HPLC chromatogram of a methanolic extract from the fruiting body of *Meripilus giganteus*, showing the peak for (1) ergothioneine.

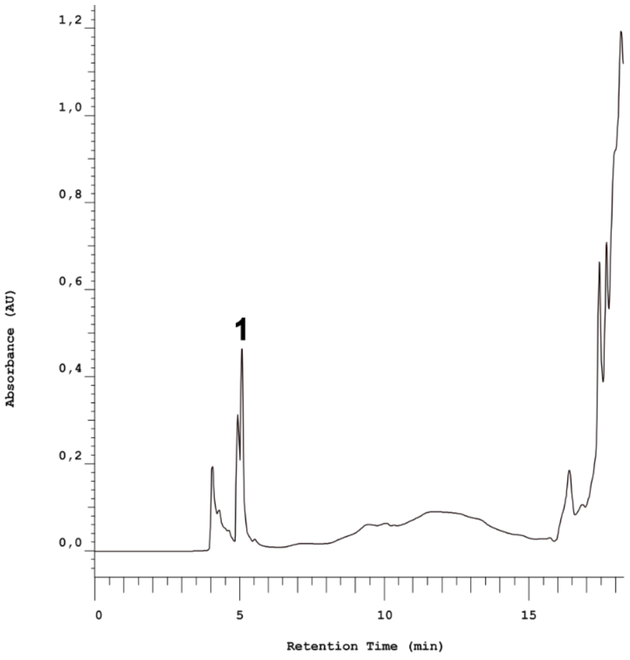

Figure S-5. Example DAD-HPLC chromatogram of an ethanolic extract from *Meripilus giganteus* mycelium, showing the peak for (1) ergothioneine.

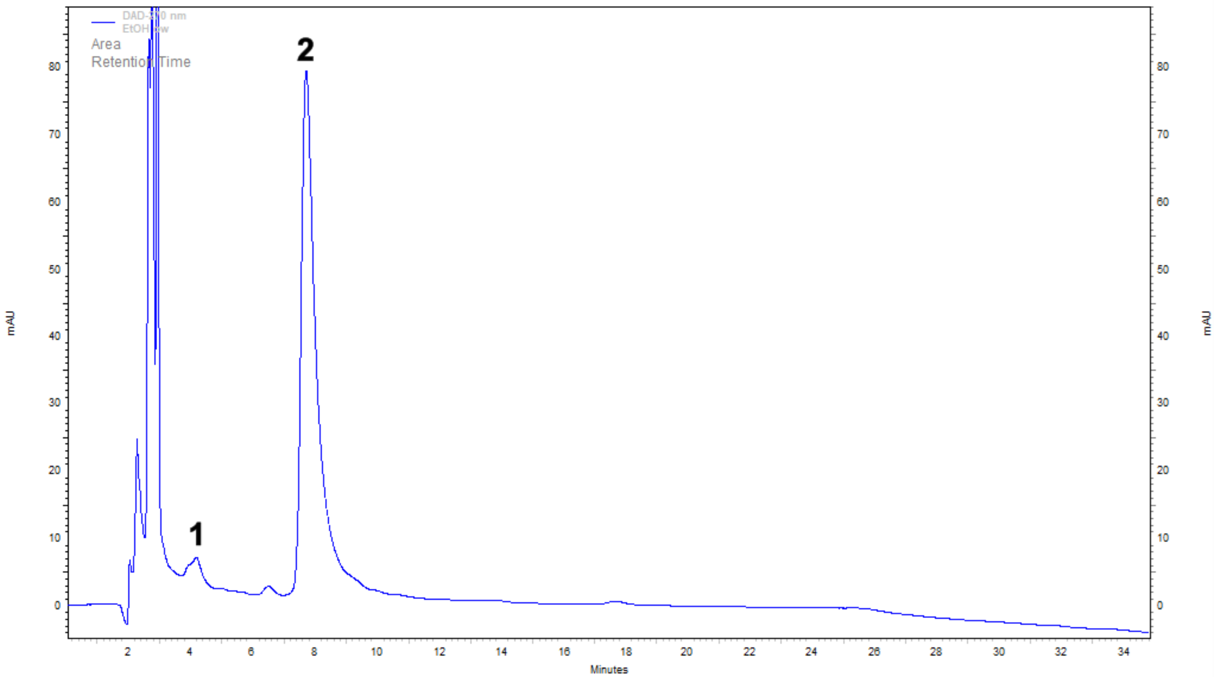

Figure S-6. Example DAD-HPLC chromatogram of an ethanolic extract from the fruiting body of *Meripilus giganteus*, showing peaks for (1) L-phenylalanine and (2) L-tryptophan.

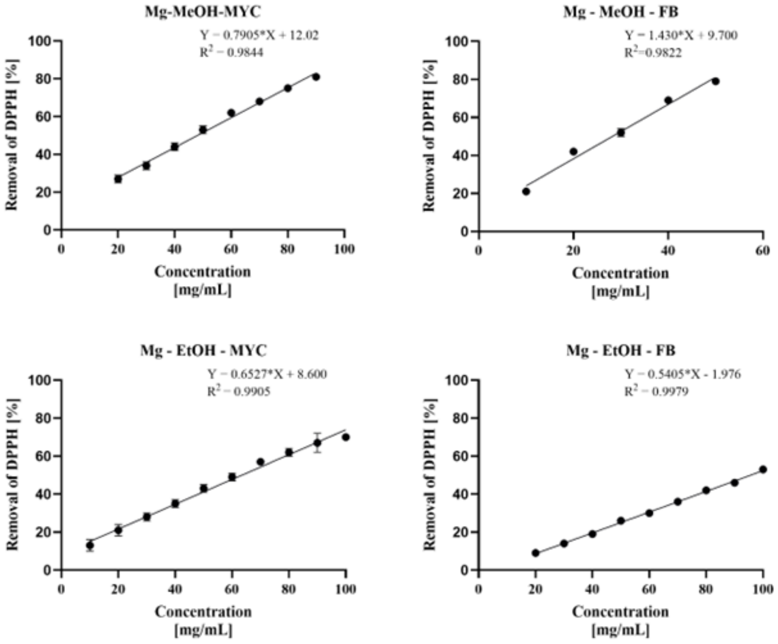

Figure S-7. DPPH radical removal (%) versus extract concentration (mg/mL) of mycelium and fruiting body extracts of *Meripilus giganteus*, depending on the solvent as mean with standard deviation, number of replicates = 3. Linear regression equations and corresponding R^2 values are presented in the graphs.

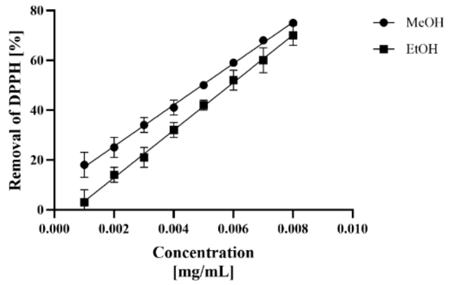

Figure S-8. DPPH radical removal (%) versus TROLOX® depending on the solvent used (MeOH/EtOH) as mean with SD, N=4. Linear regression equations were Y = 8310*X + 8,857 (R^2= 0.9988), y = 9548*x - 6,214 (R^2=0.9987) for methanol and ethanol, respectively.
